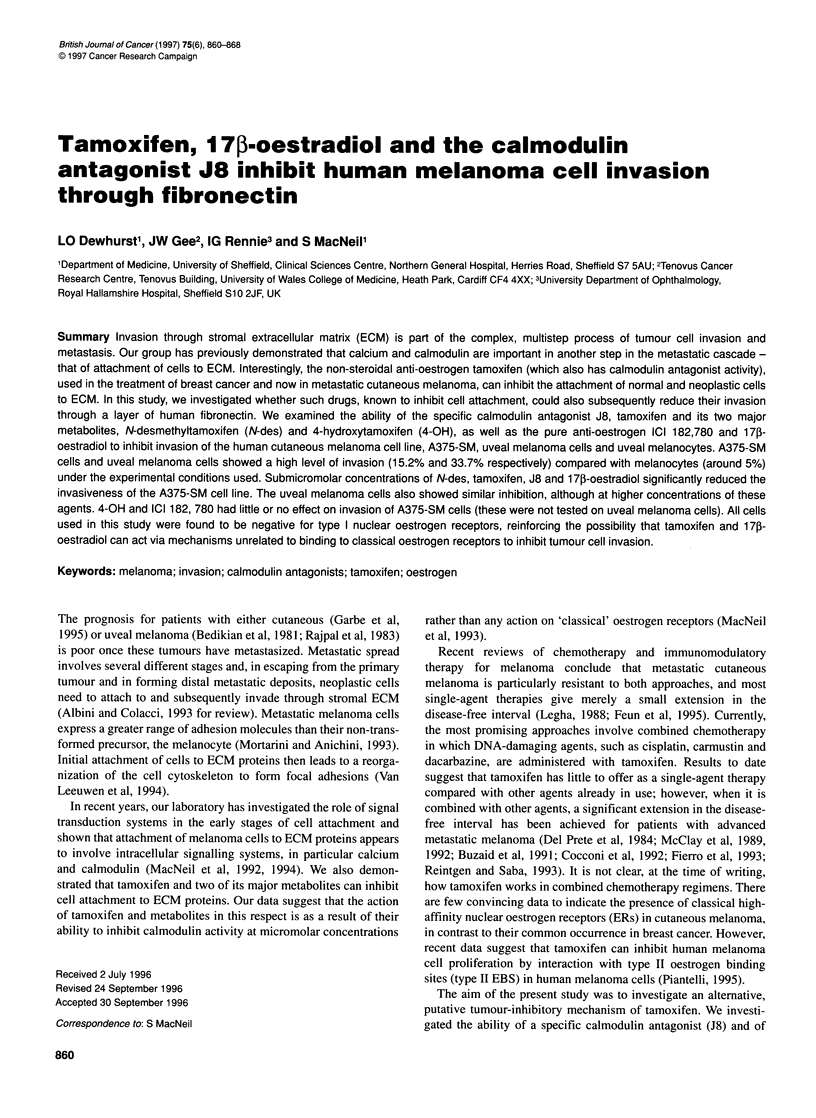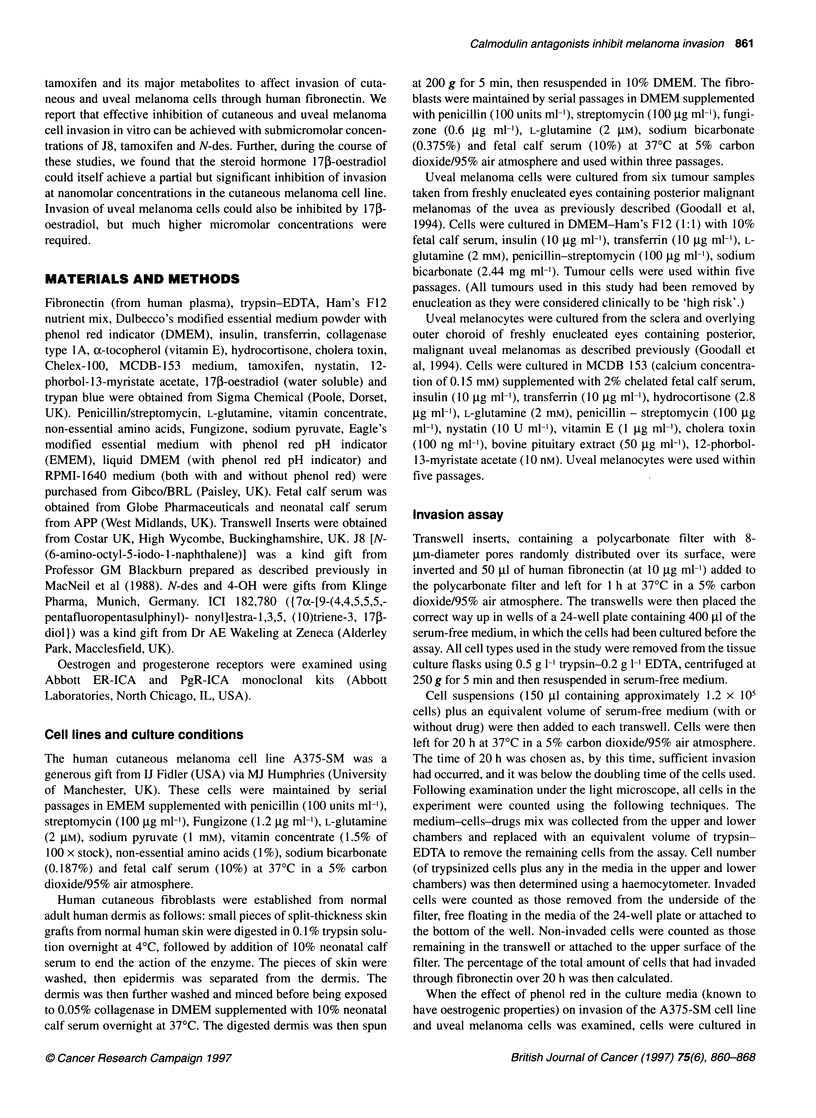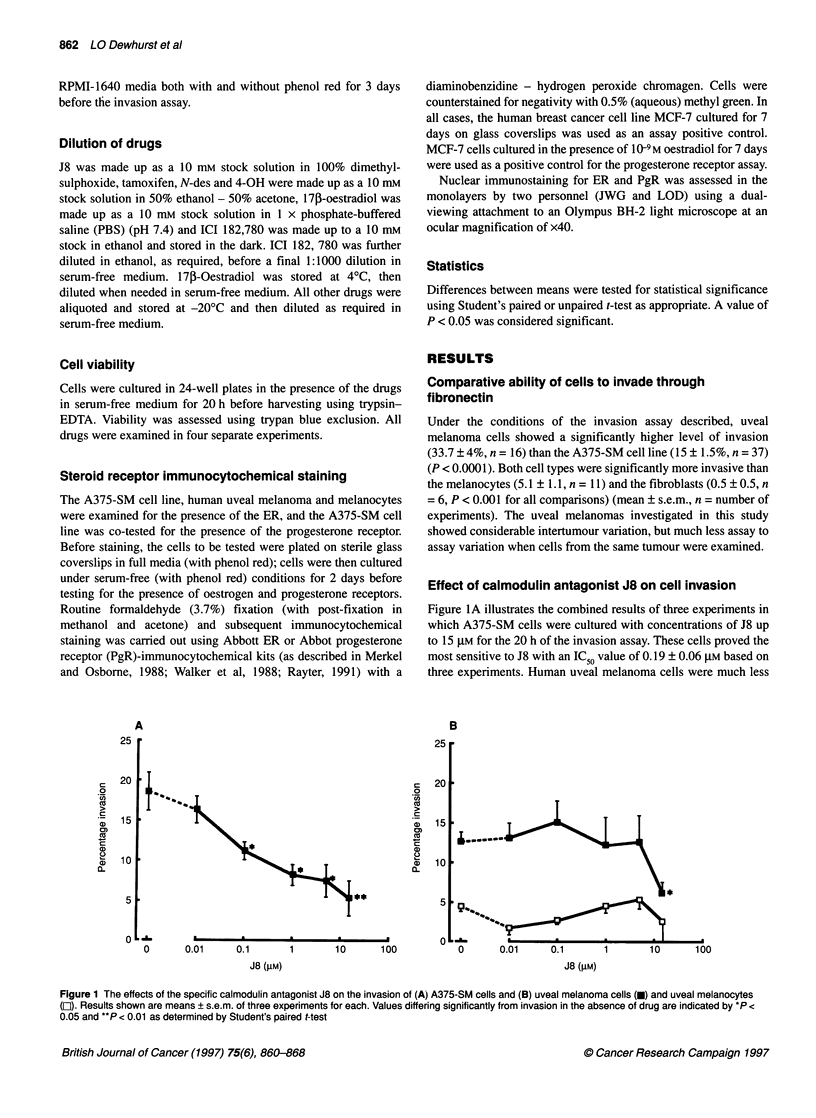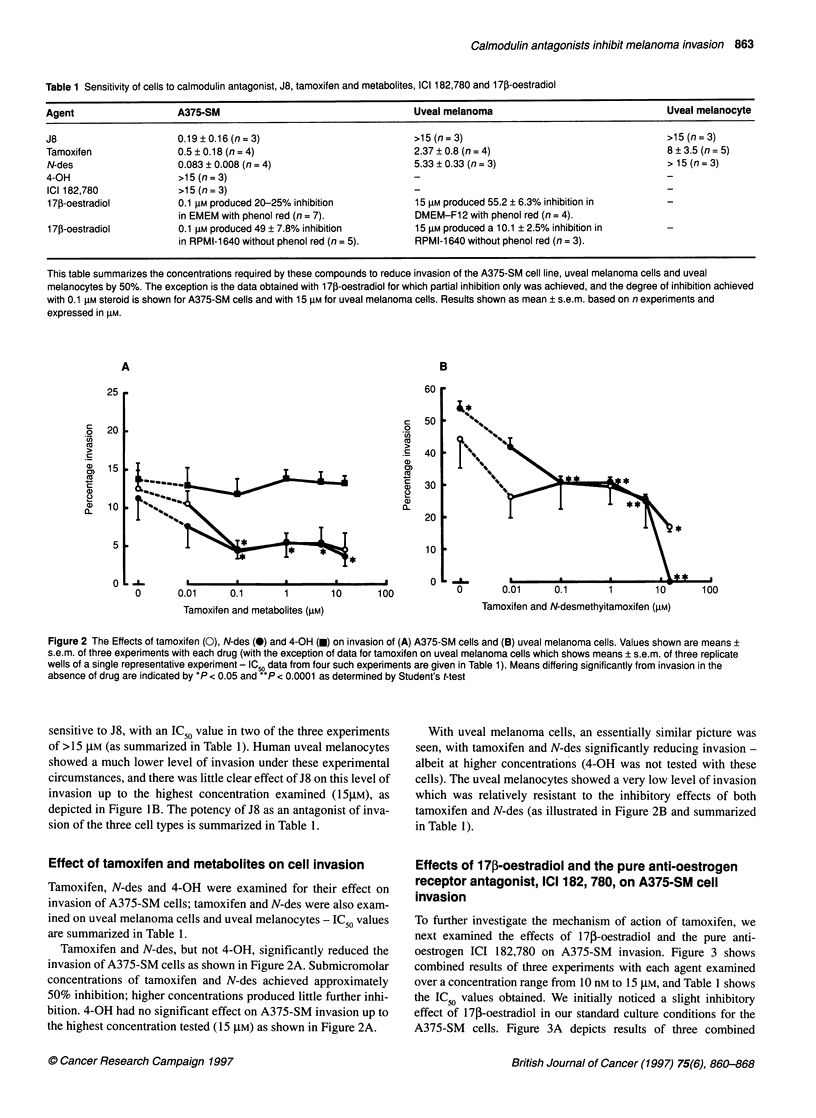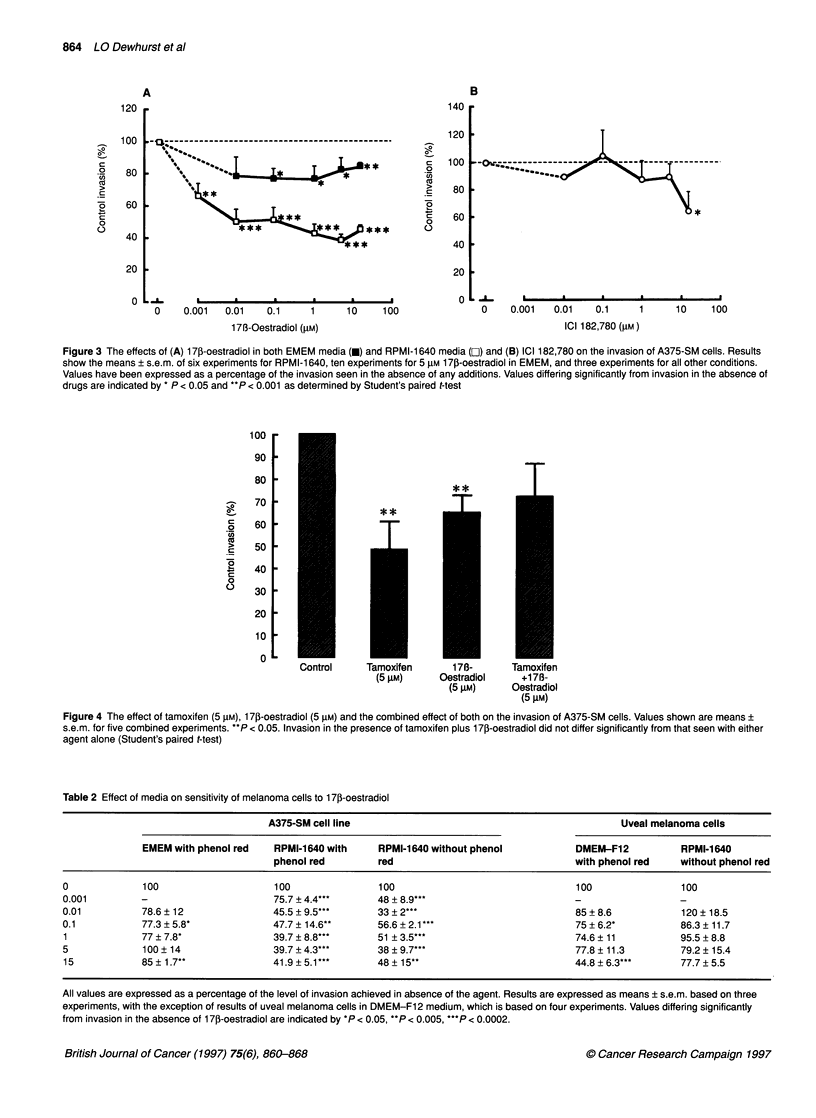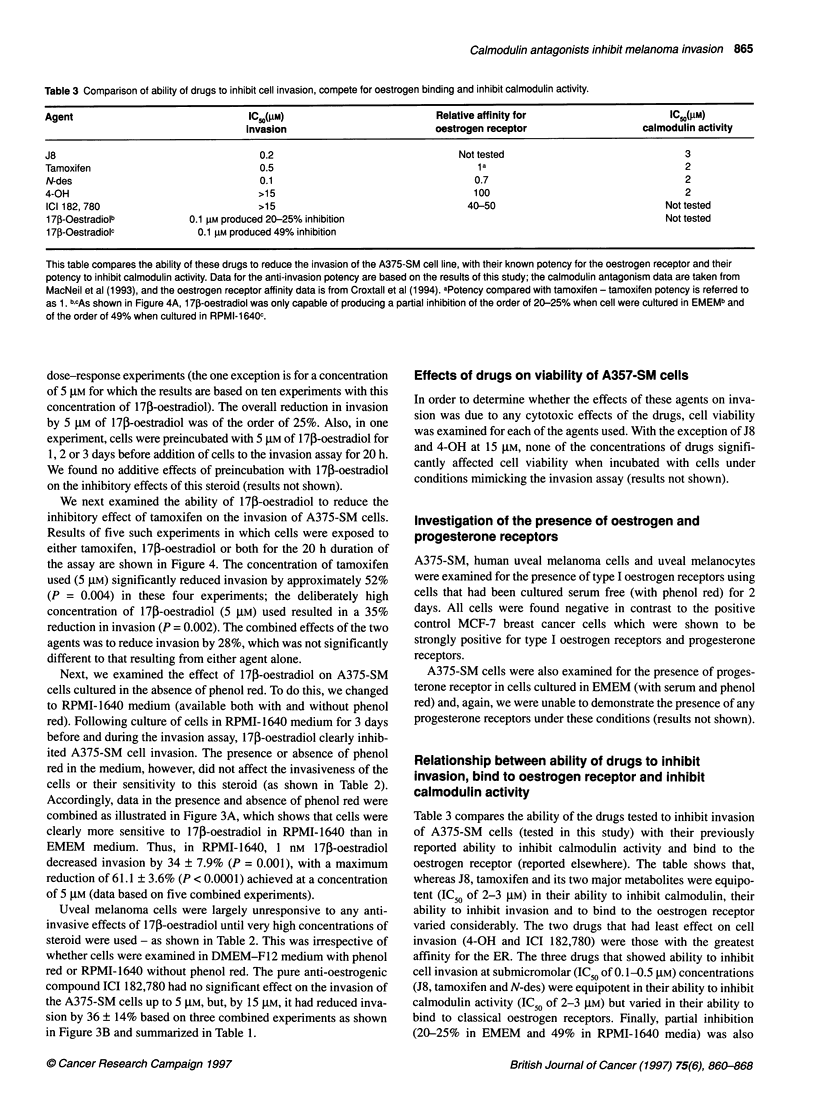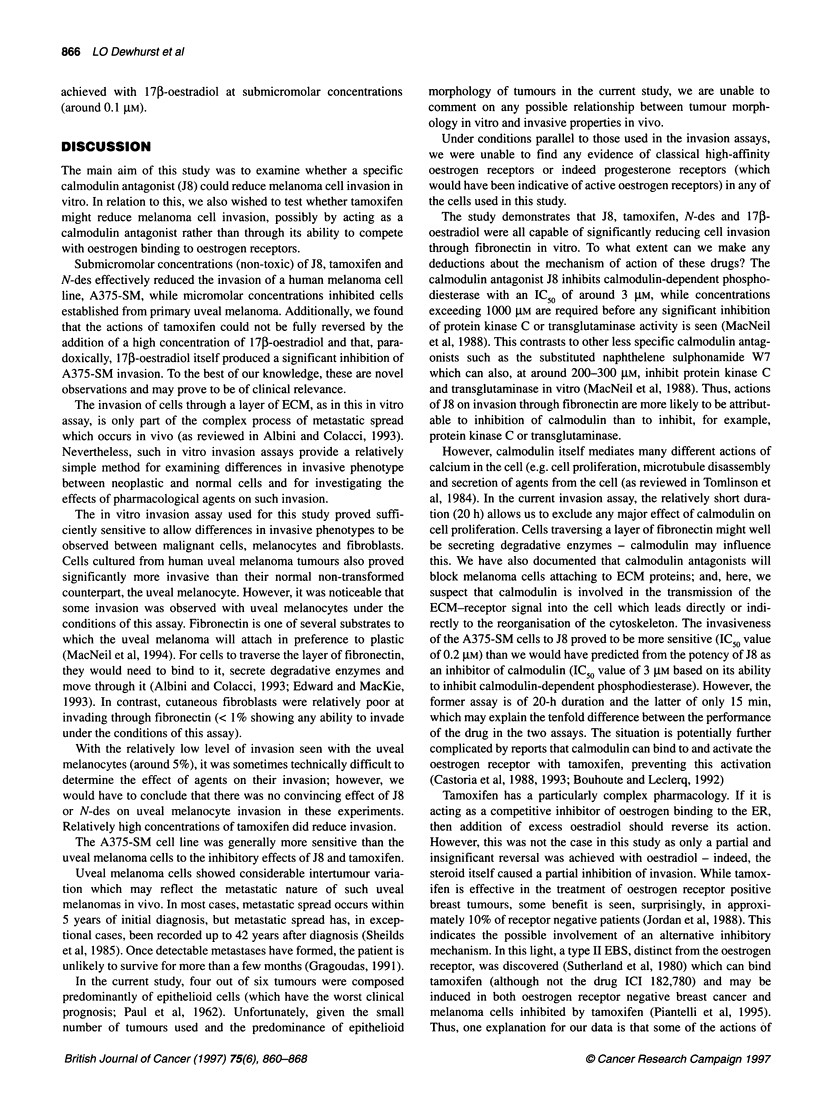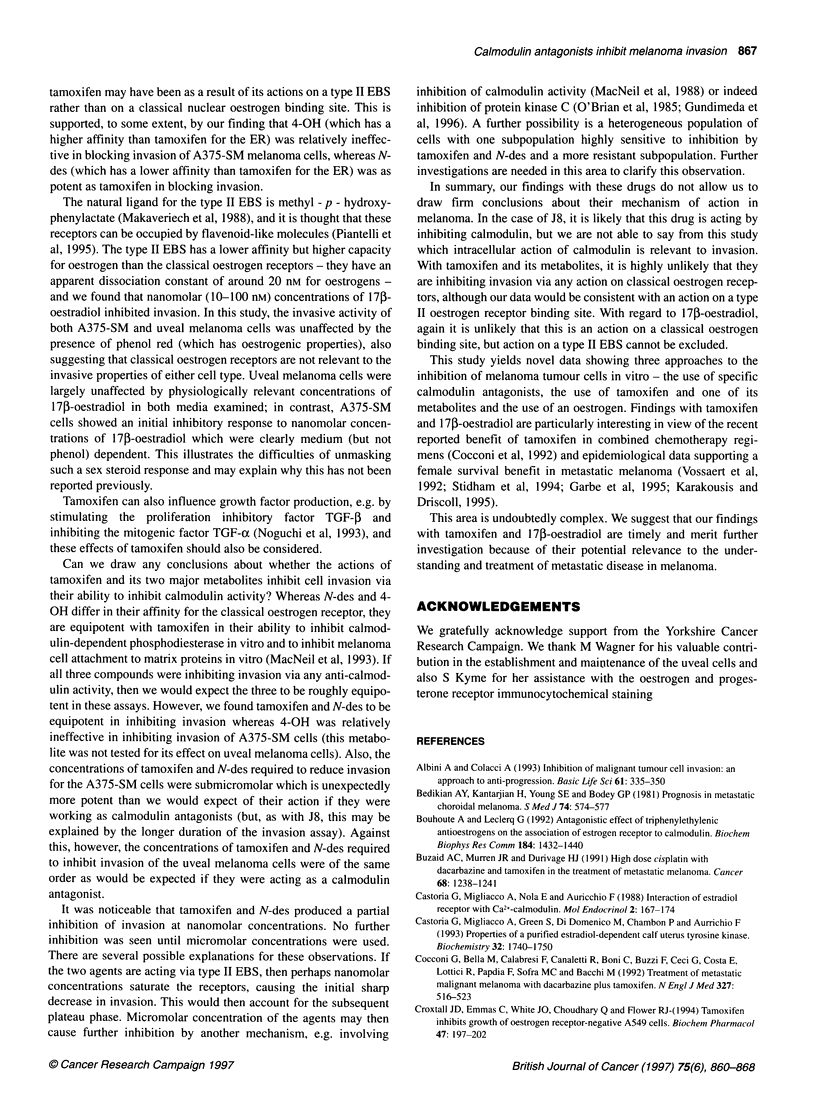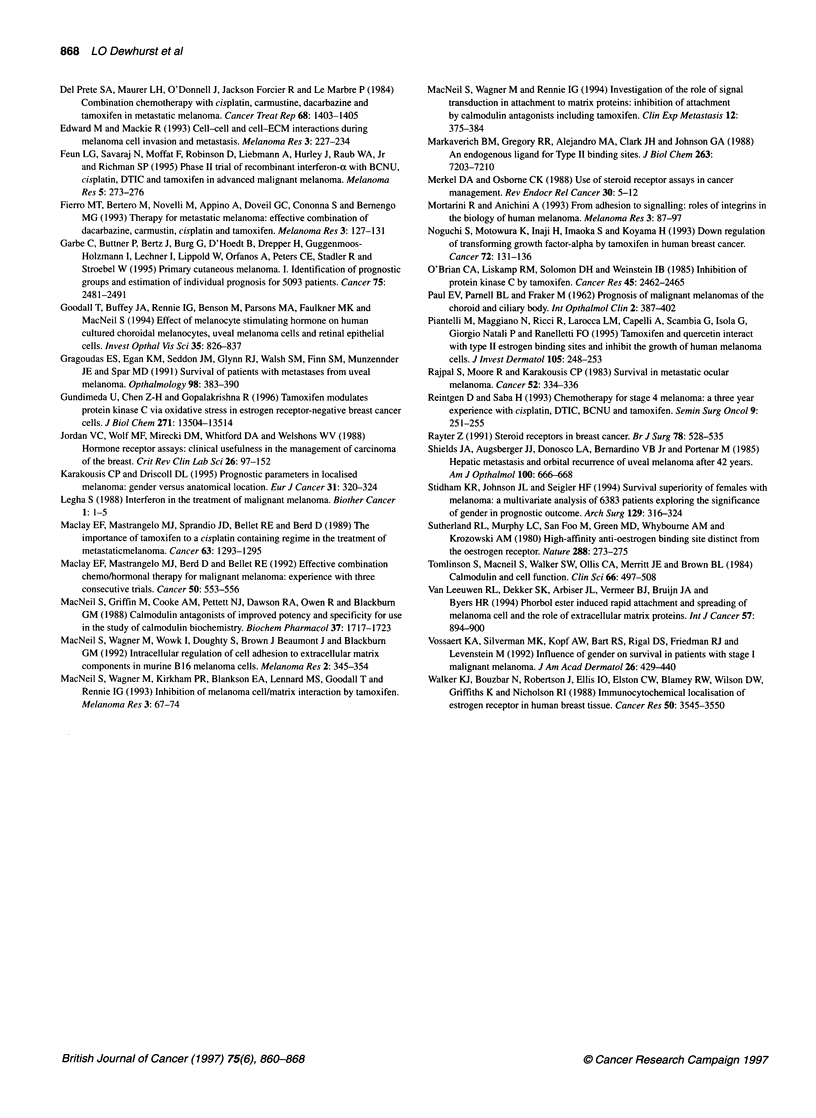# Tamoxifen, 17beta-oestradiol and the calmodulin antagonist J8 inhibit human melanoma cell invasion through fibronectin.

**DOI:** 10.1038/bjc.1997.153

**Published:** 1997

**Authors:** L. O. Dewhurst, J. W. Gee, I. G. Rennie, S. MacNeil

**Affiliations:** Department of Medicine, University of Sheffield, UK.

## Abstract

Invasion through stromal extracellular matrix (ECM) is part of the complex, multistep process of tumour cell invasion and metastasis. Our group has previously demonstrated that calcium and calmodulin are important in another step in the metastatic cascade - that of attachment of cells to ECM. Interestingly, the non-steroidal anti-oestrogen tamoxifen (which also has calmodulin antagonist activity), used in the treatment of breast cancer and now in metastatic cutaneous melanoma, can inhibit the attachment of normal and neoplastic cells to ECM. In this study, we investigated whether such drugs, known to inhibit cell attachment, could also subsequently reduce their invasion through a layer of human fibronectin. We examined the ability of the specific calmodulin antagonist J8, tamoxifen and its two major metabolites, N-desmethyltamoxifen (N-des) and 4-hydroxytamoxifen (4-OH), as well as the pure anti-oestrogen ICI 182,780 and 17beta-oestradiol to inhibit invasion of the human cutaneous melanoma cell line, A375-SM, uveal melanoma cells and uveal melanocytes. A375-SM cells and uveal melanoma cells showed a high level of invasion (15.2% and 33.7% respectively) compared with melanocytes (around 5%) under the experimental conditions used. Submicromolar concentrations of N-des, tamoxifen, J8 and 17beta-oestradiol significantly reduced the invasiveness of the A375-SM cell line. The uveal melanoma cells also showed similar inhibition, although at higher concentrations of these agents. 4-OH and ICI 182, 780 had little or no effect on invasion of A375-SM cells (these were not tested on uveal melanoma cells). All cells used in this study were found to be negative for type I nuclear oestrogen receptors, reinforcing the possibility that tamoxifen and 17beta-oestradiol can act via mechanisms unrelated to binding to classical oestrogen receptors to inhibit tumour cell invasion.


British Journal of Cancer (1997) 75(6), 860–868
© 1997 Cancer Research Campaign

### Tamoxifen, 17β-oestradiol and the calmodulin antagonist J8 inhibit human melanoma cell invasion through fibronectin

LO Dewhurst[1], JW Gee[2], IG Rennie[3] and S MacNeil[1]

[1]Department of Medicine, University of Sheffield, Clinical Sciences Centre, Northern General Hospital, Herries Road, Sheffield S7 5AU; [2]Tenovus Cancer Research Centre, Tenovus Building, University of Wales College of Medicine, Heath Park, Cardiff CF4 4XX; [3]University Department of Ophthalmology, Royal Hallamshire Hospital, Sheffield S10 2JF, UK

**Summary** Invasion through stromal extracellular matrix (ECM) is part of the complex, multistep process of tumour cell invasion and metastasis. Our group has previously demonstrated that calcium and calmodulin are important in another step in the metastatic cascade – that of attachment of cells to ECM. Interestingly, the non-steroidal anti-oestrogen tamoxifen (which also has calmodulin antagonist activity), used in the treatment of breast cancer and now in metastatic cutaneous melanoma, can inhibit the attachment of normal and neoplastic cells to ECM. In this study, we investigated whether such drugs, known to inhibit cell attachment, could also subsequently reduce their invasion through a layer of human fibronectin. We examined the ability of the specific calmodulin antagonist J8, tamoxifen and its two major metabolites, *N*-desmethyltamoxifen (*N*-des) and 4-hydroxytamoxifen (4-OH), as well as the pure anti-oestrogen ICI 182,780 and 17β-oestradiol to inhibit invasion of the human cutaneous melanoma cell line, A375-SM, uveal melanoma cells and uveal melanocytes. A375-SM cells and uveal melanoma cells showed a high level of invasion (15.2% and 33.7% respectively) compared with melanocytes (around 5%) under the experimental conditions used. Submicromolar concentrations of *N*-des, tamoxifen, J8 and 17β-oestradiol significantly reduced the invasiveness of the A375-SM cell line. The uveal melanoma cells also showed similar inhibition, although at higher concentrations of these agents. 4-OH and ICI 182, 780 had little or no effect on invasion of A375-SM cells (these were not tested on uveal melanoma cells). All cells used in this study were found to be negative for type I nuclear oestrogen receptors, reinforcing the possibility that tamoxifen and 17β-oestradiol can act via mechanisms unrelated to binding to classical oestrogen receptors to inhibit tumour cell invasion.

**Keywords:** melanoma; invasion; calmodulin antagonists; tamoxifen; oestrogen

The prognosis for patients with either cutaneous (Garbe et al, 1995) or uveal melanoma (Bedikian et al, 1981; Rajpal et al, 1983) is poor once these tumours have metastasized. Metastatic spread involves several different stages and, in escaping from the primary tumour and in forming distal metastatic deposits, neoplastic cells need to attach to and subsequently invade through stromal ECM (Albini and Colacci, 1993 for review). Metastatic melanoma cells express a greater range of adhesion molecules than their non-transformed precursor, the melanocyte (Mortarini and Anichini, 1993). Initial attachment of cells to ECM proteins then leads to a reorganization of the cell cytoskeleton to form focal adhesions (Van Leeuwen et al, 1994).

In recent years, our laboratory has investigated the role of signal transduction systems in the early stages of cell attachment and shown that attachment of melanoma cells to ECM proteins appears to involve intracellular signalling systems, in particular calcium and calmodulin (MacNeil et al, 1992, 1994). We also demonstrated that tamoxifen and two of its major metabolites can inhibit cell attachment to ECM proteins. Our data suggest that the action of tamoxifen and metabolites in this respect is as a result of their ability to inhibit calmodulin activity at micromolar concentrations rather than any action on 'classical' oestrogen receptors (MacNeil et al, 1993).

Recent reviews of chemotherapy and immunomodulatory therapy for melanoma conclude that metastatic cutaneous melanoma is particularly resistant to both approaches, and most single-agent therapies give merely a small extension in the disease-free interval (Legha, 1988; Feun et al, 1995). Currently, the most promising approaches involve combined chemotherapy in which DNA-damaging agents, such as cisplatin, carmustin and dacarbazine, are administered with tamoxifen. Results to date suggest that tamoxifen has little to offer as a single-agent therapy compared with other agents already in use; however, when it is combined with other agents, a significant extension in the disease-free interval has been achieved for patients with advanced metastatic melanoma (Del Prete et al, 1984; McClay et al, 1989, 1992; Buzaid et al, 1991; Cocconi et al, 1992; Fierro et al, 1993; Reintgen and Saba, 1993). It is not clear, at the time of writing, how tamoxifen works in combined chemotherapy regimens. There are few convincing data to indicate the presence of classical high-affinity nuclear oestrogen receptors (ERs) in cutaneous melanoma, in contrast to their common occurrence in breast cancer. However, recent data suggest that tamoxifen can inhibit human melanoma cell proliferation by interaction with type II oestrogen binding sites (type II EBS) in human melanoma cells (Piantelli, 1995).

The aim of the present study was to investigate an alternative, putative tumour-inhibitory mechanism of tamoxifen. We investigated the ability of a specific calmodulin antagonist (J8) and of

Received 2 July 1996
Revised 24 September 1996
Accepted 30 September 1996

*Correspondence to*: S MacNeil tamoxifen and its major metabolites to affect invasion of cutaneous and uveal melanoma cells through human fibronectin. We report that effective inhibition of cutaneous and uveal melanoma cell invasion in vitro can be achieved with submicromolar concentrations of J8, tamoxifen and *N*-des. Further, during the course of these studies, we found that the steroid hormone 17β-oestradiol could itself achieve a partial but significant inhibition of invasion at nanomolar concentrations in the cutaneous melanoma cell line. Invasion of uveal melanoma cells could also be inhibited by 17β-oestradiol, but much higher micromolar concentrations were required.

### MATERIALS AND METHODS

Fibronectin (from human plasma), trypsin–EDTA, Ham's F12 nutrient mix, Dulbecco's modified essential medium powder with phenol red indicator (DMEM), insulin, transferrin, collagenase type 1A, α-tocopherol (vitamin E), hydrocortisone, cholera toxin, Chelex-100, MCDB-153 medium, tamoxifen, nystatin, 12-phorbol-13-myristate acetate, 17β-oestradiol (water soluble) and trypan blue were obtained from Sigma Chemical (Poole, Dorset, UK). Penicillin/streptomycin, L-glutamine, vitamin concentrate, non-essential amino acids, Fungizone, sodium pyruvate, Eagle's modified essential medium with phenol red pH indicator (EMEM), liquid DMEM (with phenol red pH indicator) and RPMI-1640 medium (both with and without phenol red) were purchased from Gibco/BRL (Paisley, UK). Fetal calf serum was obtained from Globe Pharmaceuticals and neonatal calf serum from APP (West Midlands, UK). Transwell Inserts were obtained from Costar UK, High Wycombe, Buckinghamshire, UK. J8 [*N*-(6-amino-octyl-5-iodo-1-naphthalene)] was a kind gift from Professor GM Blackburn prepared as described previously in MacNeil et al (1988). *N*-des and 4-OH were gifts from Klinge Pharma, Munich, Germany. ICI 182,780 ({7α-[9-(4,4,5,5,5,-pentafluoropentasulphinyl)- nonyl]estra-1,3,5, (10)triene-3, 17β-diol}) was a kind gift from Dr AE Wakeling at Zeneca (Alderley Park, Macclesfield, UK).

Oestrogen and progesterone receptors were examined using Abbott ER-ICA and PgR-ICA monoclonal kits (Abbott Laboratories, North Chicago, IL, USA).

### Cell lines and culture conditions

The human cutaneous melanoma cell line A375-SM was a generous gift from IJ Fidler (USA) via MJ Humphries (University of Manchester, UK). These cells were maintained by serial passages in EMEM supplemented with penicillin (100 units $ml^{-1}$), streptomycin (100 μg $ml^{-1}$), Fungizone (1.2 μg $ml^{-1}$), L-glutamine (2 μM), sodium pyruvate (1 mM), vitamin concentrate (1.5% of 100 × stock), non-essential amino acids (1%), sodium bicarbonate (0.187%) and fetal calf serum (10%) at 37°C in a 5% carbon dioxide/95% air atmosphere.

Human cutaneous fibroblasts were established from normal adult human dermis as follows: small pieces of split-thickness skin grafts from normal human skin were digested in 0.1% trypsin solution overnight at 4°C, followed by addition of 10% neonatal calf serum to end the action of the enzyme. The pieces of skin were washed, then epidermis was separated from the dermis. The dermis was then further washed and minced before being exposed to 0.05% collagenase in DMEM supplemented with 10% neonatal calf serum overnight at 37°C. The digested dermis was then spun at 200 ***g*** for 5 min, then resuspended in 10% DMEM. The fibroblasts were maintained by serial passages in DMEM supplemented with penicillin (100 units $ml^{-1}$), streptomycin (100 μg $ml^{-1}$), fungizone (0.6 μg $ml^{-1}$), L-glutamine (2 μM), sodium bicarbonate (0.375%) and fetal calf serum (10%) at 37°C at 5% carbon dioxide/95% air atmosphere and used within three passages.

Uveal melanoma cells were cultured from six tumour samples taken from freshly enucleated eyes containing posterior malignant melanomas of the uvea as previously described (Goodall et al, 1994). Cells were cultured in DMEM–Ham's F12 (1:1) with 10% fetal calf serum, insulin (10 μg $ml^{-1}$), transferrin (10 μg $ml^{-1}$), L-glutamine (2 mM), penicillin–streptomycin (100 μg $ml^{-1}$), sodium bicarbonate (2.44 mg $ml^{-1}$). Tumour cells were used within five passages. (All tumours used in this study had been removed by enucleation as they were considered clinically to be 'high risk'.)

Uveal melanocytes were cultured from the sclera and overlying outer choroid of freshly enucleated eyes containing posterior, malignant uveal melanomas as described previously (Goodall et al, 1994). Cells were cultured in MCDB 153 (calcium concentration of 0.15 mM) supplemented with 2% chelated fetal calf serum, insulin (10 μg $ml^{-1}$), transferrin (10 μg $ml^{-1}$), hydrocortisone (2.8 μg $ml^{-1}$), L-glutamine (2 mM), penicillin – streptomycin (100 μg $ml^{-1}$), nystatin (10 U $ml^{-1}$), vitamin E (1 μg $ml^{-1}$), cholera toxin (100 ng $ml^{-1}$), bovine pituitary extract (50 μg $ml^{-1}$), 12-phorbol-13-myristate acetate (10 nM). Uveal melanocytes were used within five passages.

### Invasion assay

Transwell inserts, containing a polycarbonate filter with 8-μm-diameter pores randomly distributed over its surface, were inverted and 50 μl of human fibronectin (at 10 μg $ml^{-1}$) added to the polycarbonate filter and left for 1 h at 37°C in a 5% carbon dioxide/95% air atmosphere. The transwells were then placed the correct way up in wells of a 24-well plate containing 400 μl of the serum-free medium, in which the cells had been cultured before the assay. All cell types used in the study were removed from the tissue culture flasks using 0.5 g $l^{-1}$ trypsin–0.2 g $l^{-1}$ EDTA, centrifuged at 250 ***g*** for 5 min and then resuspended in serum-free medium.

Cell suspensions (150 μl containing approximately $1.2 \times 10^5$ cells) plus an equivalent volume of serum-free medium (with or without drug) were then added to each transwell. Cells were then left for 20 h at 37°C in a 5% carbon dioxide/95% air atmosphere. The time of 20 h was chosen as, by this time, sufficient invasion had occurred, and it was below the doubling time of the cells used. Following examination under the light microscope, all cells in the experiment were counted using the following techniques. The medium–cells–drugs mix was collected from the upper and lower chambers and replaced with an equivalent volume of trypsin–EDTA to remove the remaining cells from the assay. Cell number (of trypsinized cells plus any in the media in the upper and lower chambers) was then determined using a haemocytometer. Invaded cells were counted as those removed from the underside of the filter, free floating in the media of the 24-well plate or attached to the bottom of the well. Non-invaded cells were counted as those remaining in the transwell or attached to the upper surface of the filter. The percentage of the total amount of cells that had invaded through fibronectin over 20 h was then calculated.

When the effect of phenol red in the culture media (known to have oestrogenic properties) on invasion of the A375-SM cell line and uveal melanoma cells was examined, cells were cultured in

© Cancer Research Campaign 1997

RPMI-1640 media both with and without phenol red for 3 days before the invasion assay.

### Dilution of drugs

J8 was made up as a 10 mM stock solution in 100% dimethylsulphoxide, tamoxifen, *N*-des and 4-OH were made up as a 10 mM stock solution in 50% ethanol – 50% acetone, 17β-oestradiol was made up as a 10 mM stock solution in 1 × phosphate-buffered saline (PBS) (pH 7.4) and ICI 182,780 was made up to a 10 mM stock in ethanol and stored in the dark. ICI 182, 780 was further diluted in ethanol, as required, before a final 1:1000 dilution in serum-free medium. 17β-Oestradiol was stored at 4°C, then diluted when needed in serum-free medium. All other drugs were aliquoted and stored at –20°C and then diluted as required in serum-free medium.

### Cell viability

Cells were cultured in 24-well plates in the presence of the drugs in serum-free medium for 20 h before harvesting using trypsin–EDTA. Viability was assessed using trypan blue exclusion. All drugs were examined in four separate experiments.

### Steroid receptor immunocytochemical staining

The A375-SM cell line, human uveal melanoma and melanocytes were examined for the presence of the ER, and the A375-SM cell line was co-tested for the presence of the progesterone receptor. Before staining, the cells to be tested were plated on sterile glass coverslips in full media (with phenol red); cells were then cultured under serum-free (with phenol red) conditions for 2 days before testing for the presence of oestrogen and progesterone receptors. Routine formaldehyde (3.7%) fixation (with post-fixation in methanol and acetone) and subsequent immunocytochemical staining was carried out using Abbott ER or Abbot progesterone receptor (PgR)-immunocytochemical kits (as described in Merkel and Osborne, 1988; Walker et al, 1988; Rayter, 1991) with a diaminobenzidine – hydrogen peroxide chromagen. Cells were counterstained for negativity with 0.5% (aqueous) methyl green. In all cases, the human breast cancer cell line MCF-7 cultured for 7 days on glass coverslips was used as an assay positive control. MCF-7 cells cultured in the presence of $10^{-9}$ M oestradiol for 7 days were used as a positive control for the progesterone receptor assay.

Nuclear immunostaining for ER and PgR was assessed in the monolayers by two personnel (JWG and LOD) using a dual-viewing attachment to an Olympus BH-2 light microscope at an ocular magnification of ×40.

### Statistics

Differences between means were tested for statistical significance using Student's paired or unpaired *t*-test as appropriate. A value of $P < 0.05$ was considered significant.

### RESULTS

### Comparative ability of cells to invade through fibronectin

Under the conditions of the invasion assay described, uveal melanoma cells showed a significantly higher level of invasion (33.7 ± 4%, $n = 16$) than the A375-SM cell line (15 ± 1.5%, $n = 37$) ($P < 0.0001$). Both cell types were significantly more invasive than the melanocytes (5.1 ± 1.1, $n = 11$) and the fibroblasts (0.5 ± 0.5, $n = 6$, $P < 0.001$ for all comparisons) (mean ± s.e.m., $n$ = number of experiments). The uveal melanomas investigated in this study showed considerable intertumour variation, but much less assay to assay variation when cells from the same tumour were examined.

### Effect of calmodulin antagonist J8 on cell invasion

Figure 1A illustrates the combined results of three experiments in which A375-SM cells were cultured with concentrations of J8 up to 15 μM for the 20 h of the invasion assay. These cells proved the most sensitive to J8 with an $IC_{50}$ value of 0.19 ± 0.06 μM based on three experiments. Human uveal melanoma cells were much less

**Figure 1** The effects of the specific calmodulin antagonist J8 on the invasion of (**A**) A375-SM cells and (**B**) uveal melanoma cells (■) and uveal melanocytes (□). Results shown are means ± s.e.m. of three experiments for each. Values differing significantly from invasion in the absence of drug are indicated by $^*P < 0.05$ and $^{**}P < 0.01$ as determined by Student's paired *t*-test

© Cancer Research Campaign 1997

**Table 1** Sensitivity of cells to calmodulin antagonist, J8, tamoxifen and metabolites, ICI 182,780 and 17β-oestradiol

| Agent | A375-SM | Uveal melanoma | Uveal melanocyte |
|---|---|---|---|
| J8 | 0.19 ± 0.16 ($n$ = 3) | >15 ($n$ = 3) | >15 ($n$ = 3) |
| Tamoxifen | 0.5 ± 0.18 ($n$ = 4) | 2.37 ± 0.8 ($n$ = 4) | 8 ± 3.5 ($n$ = 5) |
| *N*-des | 0.083 ± 0.008 ($n$ = 4) | 5.33 ± 0.33 ($n$ = 3) | > 15 ($n$ = 3) |
| 4-OH | >15 ($n$ = 3) | – | – |
| ICI 182,780 | >15 ($n$ = 3) | – | – |
| 17β-oestradiol | 0.1 μM produced 20–25% inhibition in EMEM with phenol red ($n$ = 7). | 15 μM produced 55.2 ± 6.3% inhibition in DMEM–F12 with phenol red ($n$ = 4). | – |
| 17β-oestradiol | 0.1 μM produced 49 ± 7.8% inhibition in RPMI-1640 without phenol red ($n$ = 5). | 15 μM produced a 10.1 ± 2.5% inhibition in RPMI-1640 without phenol red ($n$ = 3). | – |

This table summarizes the concentrations required by these compounds to reduce invasion of the A375-SM cell line, uveal melanoma cells and uveal melanocytes by 50%. The exception is the data obtained with 17β-oestradiol for which partial inhibition only was achieved, and the degree of inhibition achieved with 0.1 μM steroid is shown for A375-SM cells and with 15 μM for uveal melanoma cells. Results shown as mean ± s.e.m. based on *n* experiments and expressed in μM.

**Figure 2** The Effects of tamoxifen (○), *N*-des (●) and 4-OH (■) on invasion of (**A**) A375-SM cells and (**B**) uveal melanoma cells. Values shown are means ± s.e.m. of three experiments with each drug (with the exception of data for tamoxifen on uveal melanoma cells which shows means ± s.e.m. of three replicate wells of a single representative experiment – $IC_{50}$ data from four such experiments are given in Table 1). Means differing significantly from invasion in the absence of drug are indicated by $^{*}P < 0.05$ and $^{**}P < 0.0001$ as determined by Student's *t*-test sensitive to J8, with an $IC_{50}$ value in two of the three experiments of >15 μM (as summarized in Table 1). Human uveal melanocytes showed a much lower level of invasion under these experimental circumstances, and there was little clear effect of J8 on this level of invasion up to the highest concentration examined (15μM), as depicted in Figure 1B. The potency of J8 as an antagonist of invasion of the three cell types is summarized in Table 1.

### Effect of tamoxifen and metabolites on cell invasion

Tamoxifen, *N*-des and 4-OH were examined for their effect on invasion of A375-SM cells; tamoxifen and *N*-des were also examined on uveal melanoma cells and uveal melanocytes – $IC_{50}$ values are summarized in Table 1.

Tamoxifen and *N*-des, but not 4-OH, significantly reduced the invasion of A375-SM cells as shown in Figure 2A. Submicromolar concentrations of tamoxifen and *N*-des achieved approximately 50% inhibition; higher concentrations produced little further inhibition. 4-OH had no significant effect on A375-SM invasion up to the highest concentration tested (15 μM) as shown in Figure 2A.

With uveal melanoma cells, an essentially similar picture was seen, with tamoxifen and *N*-des significantly reducing invasion – albeit at higher concentrations (4-OH was not tested with these cells). The uveal melanocytes showed a very low level of invasion which was relatively resistant to the inhibitory effects of both tamoxifen and *N*-des (as illustrated in Figure 2B and summarized in Table 1).

### Effects of 17β-oestradiol and the pure anti-oestrogen receptor antagonist, ICI 182, 780, on A375-SM cell invasion

To further investigate the mechanism of action of tamoxifen, we next examined the effects of 17β-oestradiol and the pure anti-oestrogen ICI 182,780 on A375-SM invasion. Figure 3 shows combined results of three experiments with each agent examined over a concentration range from 10 nM to 15 μM, and Table 1 shows the $IC_{50}$ values obtained. We initially noticed a slight inhibitory effect of 17β-oestradiol in our standard culture conditions for the A375-SM cells. Figure 3A depicts results of three combined

© Cancer Research Campaign 1997 

**Figure 3** The effects of (**A**) 17β-oestradiol in both EMEM media (■) and RPMI-1640 media (□) and (**B**) ICI 182,780 on the invasion of A375-SM cells. Results show the means ± s.e.m. of six experiments for RPMI-1640, ten experiments for 5 μM 17β-oestradiol in EMEM, and three experiments for all other conditions. Values have been expressed as a percentage of the invasion seen in the absence of any additions. Values differing significantly from invasion in the absence of drugs are indicated by * $P < 0.05$ and ** $P < 0.001$ as determined by Student's paired *t*-test

**Figure 4** The effect of tamoxifen (5 μM), 17β-oestradiol (5 μM) and the combined effect of both on the invasion of A375-SM cells. Values shown are means ± s.e.m. for five combined experiments. ** $P < 0.05$. Invasion in the presence of tamoxifen plus 17β-oestradiol did not differ significantly from that seen with either agent alone (Student's paired *t*-test)

**Table 2** Effect of media on sensitivity of melanoma cells to 17β-oestradiol

| | A375-SM cell line | | | Uveal melanoma cells | |
|---|---|---|---|---|---|
| | **EMEM with phenol red** | **RPMI-1640 with phenol red** | **RPMI-1640 without phenol red** | **DMEM–F12 with phenol red** | **RPMI-1640 without phenol red** |
| 0 | 100 | 100 | 100 | 100 | 100 |
| 0.001 | – | 75.7 ± 4.4*** | 48 ± 8.9*** | – | – |
| 0.01 | 78.6 ± 12 | 45.5 ± 9.5*** | 33 ± 2*** | 85 ± 8.6 | 120 ± 18.5 |
| 0.1 | 77.3 ± 5.8* | 47.7 ± 14.6** | 56.6 ± 2.1*** | 75 ± 6.2* | 86.3 ± 11.7 |
| 1 | 77 ± 7.8* | 39.7 ± 8.8*** | 51 ± 3.5*** | 74.6 ± 11 | 95.5 ± 8.8 |
| 5 | 100 ± 14 | 39.7 ± 4.3*** | 38 ± 9.7*** | 77.8 ± 11.3 | 79.2 ± 15.4 |
| 15 | 85 ± 1.7** | 41.9 ± 5.1*** | 48 ± 15** | 44.8 ± 6.3*** | 77.7 ± 5.5 |

All values are expressed as a percentage of the level of invasion achieved in absence of the agent. Results are expressed as means ± s.e.m. based on three experiments, with the exception of results of uveal melanoma cells in DMEM–F12 medium, which is based on four experiments. Values differing significantly from invasion in the absence of 17β-oestradiol are indicated by * $P < 0.05$, ** $P < 0.005$, *** $P < 0.0002$.

 © Cancer Research Campaign 1997

**Table 3** Comparison of ability of drugs to inhibit cell invasion, compete for oestrogen binding and inhibit calmodulin activity.

| Agent | $IC_{50}$(μM) invasion | Relative affinity for oestrogen receptor | $IC_{50}$(μM) calmodulin activity |
|---|---|---|---|
| J8 | 0.2 | Not tested | 3 |
| Tamoxifen | 0.5 | 1[a] | 2 |
| *N*-des | 0.1 | 0.7 | 2 |
| 4-OH | >15 | 100 | 2 |
| ICI 182, 780 | >15 | 40–50 | Not tested |
| 17β-Oestradiol[b] | 0.1 μM produced 20–25% inhibition | | Not tested |
| 17β-Oestradiol[c] | 0.1 μM produced 49% inhibition | | |

This table compares the ability of these drugs to reduce the invasion of the A375-SM cell line, with their known potency for the oestrogen receptor and their potency to inhibit calmodulin activity. Data for the anti-invasion potency are based on the results of this study; the calmodulin antagonism data are taken from MacNeil et al (1993), and the oestrogen receptor affinity data is from Croxtall et al (1994). [a]Potency compared with tamoxifen – tamoxifen potency is referred to as 1. [b,c]As shown in Figure 4A, 17β-oestradiol was only capable of producing a partial inhibition of the order of 20–25% when cell were cultured in EMEM[b] and of the order of 49% when cultured in RPMI-1640[c].

dose–response experiments (the one exception is for a concentration of 5 μM for which the results are based on ten experiments with this concentration of 17β-oestradiol). The overall reduction in invasion by 5 μM of 17β-oestradiol was of the order of 25%. Also, in one experiment, cells were preincubated with 5 μM of 17β-oestradiol for 1, 2 or 3 days before addition of cells to the invasion assay for 20 h. We found no additive effects of preincubation with 17β-oestradiol on the inhibitory effects of this steroid (results not shown).

We next examined the ability of 17β-oestradiol to reduce the inhibitory effect of tamoxifen on the invasion of A375-SM cells. Results of five such experiments in which cells were exposed to either tamoxifen, 17β-oestradiol or both for the 20 h duration of the assay are shown in Figure 4. The concentration of tamoxifen used (5 μM) significantly reduced invasion by approximately 52% ($P$ = 0.004) in these four experiments; the deliberately high concentration of 17β-oestradiol (5 μM) used resulted in a 35% reduction in invasion ($P = 0.002$). The combined effects of the two agents was to reduce invasion by 28%, which was not significantly different to that resulting from either agent alone.

Next, we examined the effect of 17β-oestradiol on A375-SM cells cultured in the absence of phenol red. To do this, we changed to RPMI-1640 medium (available both with and without phenol red). Following culture of cells in RPMI-1640 medium for 3 days before and during the invasion assay, 17β-oestradiol clearly inhibited A375-SM cell invasion. The presence or absence of phenol red in the medium, however, did not affect the invasiveness of the cells or their sensitivity to this steroid (as shown in Table 2). Accordingly, data in the presence and absence of phenol red were combined as illustrated in Figure 3A, which shows that cells were clearly more sensitive to 17β-oestradiol in RPMI-1640 than in EMEM medium. Thus, in RPMI-1640, 1 nM 17β-oestradiol decreased invasion by 34 ± 7.9% ($P$ = 0.001), with a maximum reduction of 61.1 ± 3.6% ($P < 0.0001$) achieved at a concentration of 5 μM (data based on five combined experiments).

Uveal melanoma cells were largely unresponsive to any anti-invasive effects of 17β-oestradiol until very high concentrations of steroid were used – as shown in Table 2. This was irrespective of whether cells were examined in DMEM–F12 medium with phenol red or RPMI-1640 without phenol red. The pure anti-oestrogenic compound ICI 182,780 had no significant effect on the invasion of the A375-SM cells up to 5 μM, but, by 15 μM, it had reduced invasion by 36 ± 14% based on three combined experiments as shown in Figure 3B and summarized in Table 1.

### Effects of drugs on viability of A357-SM cells

In order to determine whether the effects of these agents on invasion was due to any cytotoxic effects of the drugs, cell viability was examined for each of the agents used. With the exception of J8 and 4-OH at 15 μM, none of the concentrations of drugs significantly affected cell viability when incubated with cells under conditions mimicking the invasion assay (results not shown).

### Investigation of the presence of oestrogen and progesterone receptors

A375-SM, human uveal melanoma cells and uveal melanocytes were examined for the presence of type I oestrogen receptors using cells that had been cultured serum free (with phenol red) for 2 days. All cells were found negative in contrast to the positive control MCF-7 breast cancer cells which were shown to be strongly positive for type I oestrogen receptors and progesterone receptors.

A375-SM cells were also examined for the presence of progesterone receptor in cells cultured in EMEM (with serum and phenol red) and, again, we were unable to demonstrate the presence of any progesterone receptors under these conditions (results not shown).

### Relationship between ability of drugs to inhibit invasion, bind to oestrogen receptor and inhibit calmodulin activity

Table 3 compares the ability of the drugs tested to inhibit invasion of A375-SM cells (tested in this study) with their previously reported ability to inhibit calmodulin activity and bind to the oestrogen receptor (reported elsewhere). The table shows that, whereas J8, tamoxifen and its two major metabolites were equipotent ($IC_{50}$ of 2–3 μM) in their ability to inhibit calmodulin, their ability to inhibit invasion and to bind to the oestrogen receptor varied considerably. The two drugs that had least effect on cell invasion (4-OH and ICI 182,780) were those with the greatest affinity for the ER. The three drugs that showed ability to inhibit cell invasion at submicromolar ($IC_{50}$ of 0.1–0.5 μM) concentrations (J8, tamoxifen and *N*-des) were equipotent in their ability to inhibit calmodulin activity ($IC_{50}$ of 2–3 μM) but varied in their ability to bind to classical oestrogen receptors. Finally, partial inhibition (20–25% in EMEM and 49% in RPMI-1640 media) was also

© Cancer Research Campaign 1997

achieved with 17β-oestradiol at submicromolar concentrations (around 0.1 μM).

### DISCUSSION

The main aim of this study was to examine whether a specific calmodulin antagonist (J8) could reduce melanoma cell invasion in vitro. In relation to this, we also wished to test whether tamoxifen might reduce melanoma cell invasion, possibly by acting as a calmodulin antagonist rather than through its ability to compete with oestrogen binding to oestrogen receptors.

Submicromolar concentrations (non-toxic) of J8, tamoxifen and *N*-des effectively reduced the invasion of a human melanoma cell line, A375-SM, while micromolar concentrations inhibited cells established from primary uveal melanoma. Additionally, we found that the actions of tamoxifen could not be fully reversed by the addition of a high concentration of 17β-oestradiol and that, paradoxically, 17β-oestradiol itself produced a significant inhibition of A375-SM invasion. To the best of our knowledge, these are novel observations and may prove to be of clinical relevance.

The invasion of cells through a layer of ECM, as in this in vitro assay, is only part of the complex process of metastatic spread which occurs in vivo (as reviewed in Albini and Colacci, 1993). Nevertheless, such in vitro invasion assays provide a relatively simple method for examining differences in invasive phenotype between neoplastic and normal cells and for investigating the effects of pharmacological agents on such invasion.

The in vitro invasion assay used for this study proved sufficiently sensitive to allow differences in invasive phenotypes to be observed between malignant cells, melanocytes and fibroblasts. Cells cultured from human uveal melanoma tumours also proved significantly more invasive than their normal non-transformed counterpart, the uveal melanocyte. However, it was noticeable that some invasion was observed with uveal melanocytes under the conditions of this assay. Fibronectin is one of several substrates to which the uveal melanoma will attach in preference to plastic (MacNeil et al, 1994). For cells to traverse the layer of fibronectin, they would need to bind to it, secrete degradative enzymes and move through it (Albini and Colacci, 1993; Edward and MacKie, 1993). In contrast, cutaneous fibroblasts were relatively poor at invading through fibronectin (< 1% showing any ability to invade under the conditions of this assay).

With the relatively low level of invasion seen with the uveal melanocytes (around 5%), it was sometimes technically difficult to determine the effect of agents on their invasion; however, we would have to conclude that there was no convincing effect of J8 or *N*-des on uveal melanocyte invasion in these experiments. Relatively high concentrations of tamoxifen did reduce invasion.

The A375-SM cell line was generally more sensitive than the uveal melanoma cells to the inhibitory effects of J8 and tamoxifen.

Uveal melanoma cells showed considerable intertumour variation which may reflect the metastatic nature of such uveal melanomas in vivo. In most cases, metastatic spread occurs within 5 years of initial diagnosis, but metastatic spread has, in exceptional cases, been recorded up to 42 years after diagnosis (Sheilds et al, 1985). Once detectable metastases have formed, the patient is unlikely to survive for more than a few months (Gragoudas, 1991).

In the current study, four out of six tumours were composed predominantly of epithelioid cells (which have the worst clinical prognosis; Paul et al, 1962). Unfortunately, given the small number of tumours used and the predominance of epithelioid morphology of tumours in the current study, we are unable to comment on any possible relationship between tumour morphology in vitro and invasive properties in vivo.

Under conditions parallel to those used in the invasion assays, we were unable to find any evidence of classical high-affinity oestrogen receptors or indeed progesterone receptors (which would have been indicative of active oestrogen receptors) in any of the cells used in this study.

The study demonstrates that J8, tamoxifen, *N*-des and 17β-oestradiol were all capable of significantly reducing cell invasion through fibronectin in vitro. To what extent can we make any deductions about the mechanism of action of these drugs? The calmodulin antagonist J8 inhibits calmodulin-dependent phosphodiesterase with an $IC_{50}$ of around 3 μM, while concentrations exceeding 1000 μM are required before any significant inhibition of protein kinase C or transglutaminase activity is seen (MacNeil et al, 1988). This contrasts to other less specific calmodulin antagonists such as the substituted naphthelene sulphonamide W7 which can also, at around 200–300 μM, inhibit protein kinase C and transglutaminase in vitro (MacNeil et al, 1988). Thus, actions of J8 on invasion through fibronectin are more likely to be attributable to inhibition of calmodulin than to inhibit, for example, protein kinase C or transglutaminase.

However, calmodulin itself mediates many different actions of calcium in the cell (e.g. cell proliferation, microtubule disassembly and secretion of agents from the cell (as reviewed in Tomlinson et al, 1984). In the current invasion assay, the relatively short duration (20 h) allows us to exclude any major effect of calmodulin on cell proliferation. Cells traversing a layer of fibronectin might well be secreting degradative enzymes – calmodulin may influence this. We have also documented that calmodulin antagonists will block melanoma cells attaching to ECM proteins; and, here, we suspect that calmodulin is involved in the transmission of the ECM–receptor signal into the cell which leads directly or indirectly to the reorganisation of the cytoskeleton. The invasiveness of the A375-SM cells to J8 proved to be more sensitive ($IC_{50}$ value of 0.2 μM) than we would have predicted from the potency of J8 as an inhibitor of calmodulin ($IC_{50}$ value of 3 μM based on its ability to inhibit calmodulin-dependent phosphodiesterase). However, the former assay is of 20-h duration and the latter of only 15 min, which may explain the tenfold difference between the performance of the drug in the two assays. The situation is potentially further complicated by reports that calmodulin can bind to and activate the oestrogen receptor with tamoxifen, preventing this activation (Castoria et al, 1988, 1993; Bouhoute and Leclerq, 1992)

Tamoxifen has a particularly complex pharmacology. If it is acting as a competitive inhibitor of oestrogen binding to the ER, then addition of excess oestradiol should reverse its action. However, this was not the case in this study as only a partial and insignificant reversal was achieved with oestradiol – indeed, the steroid itself caused a partial inhibition of invasion. While tamoxifen is effective in the treatment of oestrogen receptor positive breast tumours, some benefit is seen, surprisingly, in approximately 10% of receptor negative patients (Jordan et al, 1988). This indicates the possible involvement of an alternative inhibitory mechanism. In this light, a type II EBS, distinct from the oestrogen receptor, was discovered (Sutherland et al, 1980) which can bind tamoxifen (although not the drug ICI 182,780) and may be induced in both oestrogen receptor negative breast cancer and melanoma cells inhibited by tamoxifen (Piantelli et al, 1995). Thus, one explanation for our data is that some of the actions of

© Cancer Research Campaign 1997

tamoxifen may have been as a result of its actions on a type II EBS rather than on a classical nuclear oestrogen binding site. This is supported, to some extent, by our finding that 4-OH (which has a higher affinity than tamoxifen for the ER) was relatively ineffective in blocking invasion of A375-SM melanoma cells, whereas *N*-des (which has a lower affinity than tamoxifen for the ER) was as potent as tamoxifen in blocking invasion.

The natural ligand for the type II EBS is methyl - *p* - hydroxyphenylactate (Makaveriech et al, 1988), and it is thought that these receptors can be occupied by flavenoid-like molecules (Piantelli et al, 1995). The type II EBS has a lower affinity but higher capacity for oestrogen than the classical oestrogen receptors – they have an apparent dissociation constant of around 20 nM for oestrogens – and we found that nanomolar (10–100 nM) concentrations of 17β-oestradiol inhibited invasion. In this study, the invasive activity of both A375-SM and uveal melanoma cells was unaffected by the presence of phenol red (which has oestrogenic properties), also suggesting that classical oestrogen receptors are not relevant to the invasive properties of either cell type. Uveal melanoma cells were largely unaffected by physiologically relevant concentrations of 17β-oestradiol in both media examined; in contrast, A375-SM cells showed an initial inhibitory response to nanomolar concentrations of 17β-oestradiol which were clearly medium (but not phenol) dependent. This illustrates the difficulties of unmasking such a sex steroid response and may explain why this has not been reported previously.

Tamoxifen can also influence growth factor production, e.g. by stimulating the proliferation inhibitory factor TGF-β and inhibiting the mitogenic factor TGF-α (Noguchi et al, 1993), and these effects of tamoxifen should also be considered.

Can we draw any conclusions about whether the actions of tamoxifen and its two major metabolites inhibit cell invasion via their ability to inhibit calmodulin activity? Whereas *N*-des and 4-OH differ in their affinity for the classical oestrogen receptor, they are equipotent with tamoxifen in their ability to inhibit calmodulin-dependent phosphodiesterase in vitro and to inhibit melanoma cell attachment to matrix proteins in vitro (MacNeil et al, 1993). If all three compounds were inhibiting invasion via any anti-calmodulin activity, then we would expect the three to be roughly equipotent in these assays. However, we found tamoxifen and *N*-des to be equipotent in inhibiting invasion whereas 4-OH was relatively ineffective in inhibiting invasion of A375-SM cells (this metabolite was not tested for its effect on uveal melanoma cells). Also, the concentrations of tamoxifen and *N*-des required to reduce invasion for the A375-SM cells were submicromolar which is unexpectedly more potent than we would expect of their action if they were working as calmodulin antagonists (but, as with J8, this may be explained by the longer duration of the invasion assay). Against this, however, the concentrations of tamoxifen and *N*-des required to inhibit invasion of the uveal melanoma cells were of the same order as would be expected if they were acting as a calmodulin antagonist.

It was noticeable that tamoxifen and *N*-des produced a partial inhibition of invasion at nanomolar concentrations. No further inhibition was seen until micromolar concentrations were used. There are several possible explanations for these observations. If the two agents are acting via type II EBS, then perhaps nanomolar concentrations saturate the receptors, causing the initial sharp decrease in invasion. This would then account for the subsequent plateau phase. Micromolar concentration of the agents may then cause further inhibition by another mechanism, e.g. involving inhibition of calmodulin activity (MacNeil et al, 1988) or indeed inhibition of protein kinase C (O'Brian et al, 1985; Gundimeda et al, 1996). A further possibility is a heterogeneous population of cells with one subpopulation highly sensitive to inhibition by tamoxifen and *N*-des and a more resistant subpopulation. Further investigations are needed in this area to clarify this observation.

In summary, our findings with these drugs do not allow us to draw firm conclusions about their mechanism of action in melanoma. In the case of J8, it is likely that this drug is acting by inhibiting calmodulin, but we are not able to say from this study which intracellular action of calmodulin is relevant to invasion. With tamoxifen and its metabolites, it is highly unlikely that they are inhibiting invasion via any action on classical oestrogen receptors, although our data would be consistent with an action on a type II oestrogen receptor binding site. With regard to 17β-oestradiol, again it is unlikely that this is an action on a classical oestrogen binding site, but action on a type II EBS cannot be excluded.

This study yields novel data showing three approaches to the inhibition of melanoma tumour cells in vitro – the use of specific calmodulin antagonists, the use of tamoxifen and one of its metabolites and the use of an oestrogen. Findings with tamoxifen and 17β-oestradiol are particularly interesting in view of the recent reported benefit of tamoxifen in combined chemotherapy regimens (Cocconi et al, 1992) and epidemiological data supporting a female survival benefit in metastatic melanoma (Vossaert et al, 1992; Stidham et al, 1994; Garbe et al, 1995; Karakousis and Driscoll, 1995).

This area is undoubtedly complex. We suggest that our findings with tamoxifen and 17β-oestradiol are timely and merit further investigation because of their potential relevance to the understanding and treatment of metastatic disease in melanoma.

### ACKNOWLEDGEMENTS

We gratefully acknowledge support from the Yorkshire Cancer Research Campaign. We thank M Wagner for his valuable contribution in the establishment and maintenance of the uveal cells and also S Kyme for her assistance with the oestrogen and progesterone receptor immunocytochemical staining

### REFERENCES

Albini A and Colacci A (1993) Inhibition of malignant tumour cell invasion: an approach to anti-progression. *Basic Life Sci* **61**: 335–350

Bedikian AY, Kantarjian H, Young SE and Bodey GP (1981) Prognosis in metastatic choroidal melanoma. *S Med J* **74**: 574–577

Bouhoute A and Leclerq G (1992) Antagonistic effect of triphenylethylenic antioestrogens on the association of estrogen receptor to calmodulin. *Biochem Biophys Res Comm* **184**: 1432–1440

Buzaid AC, Murren JR and Durivage HJ (1991) High dose *cis*platin with dacarbazine and tamoxifen in the treatment of metastatic melanoma. *Cancer* **68**: 1238–1241

Castoria G, Migliacco A, Nola E and Auricchio F (1988) Interaction of estradiol receptor with $Ca^{2+}$-calmodulin. *Mol Endocrinol* **2**: 167–174

Castoria G, Migliacco A, Green S, Di Domenico M, Chambon P and Aurricchio F (1993) Properties of a purified estradiol-dependent calf uterus tyrosine kinase. *Biochemistry* **32**: 1740–1750

Cocconi G, Bella M, Calabresi F, Canaletti R, Boni C, Buzzi F, Ceci G, Costa E, Lottici R, Papdia F, Sofra MC and Bacchi M (1992) Treatment of metastatic malignant melanoma with dacarbazine plus tamoxifen. *N Engl J Med* **327**: 516–523

Croxtall JD, Emmas C, White JO, Choudhary Q and Flower RJ-(1994) Tamoxifen inhibits growth of oestrogen receptor-negative A549 cells. *Biochem Pharmacol* **47**: 197–202

© Cancer Research Campaign 1997

Del Prete SA, Maurer LH, O'Donnell J, Jackson Forcier R and Le Marbre P (1984) Combination chemotherapy with *cis*platin, carmustine, dacarbazine and tamoxifen in metastatic melanoma. *Cancer Treat Rep* **68**: 1403–1405

Edward M and Mackie R (1993) Cell–cell and cell–ECM interactions during melanoma cell invasion and metastasis. *Melanoma Res* **3**: 227–234

Feun LG, Savaraj N, Moffat F, Robinson D, Liebmann A, Hurley J, Raub WA, Jr and Richman SP (1995) Phase II trial of recombinant interferon-α with BCNU, *cis*platin, DTIC and tamoxifen in advanced malignant melanoma. *Melanoma Res* **5**: 273–276

Fierro MT, Bertero M, Novelli M, Appino A, Doveil GC, Cononna S and Bernengo MG (1993) Therapy for metastatic melanoma: effective combination of dacarbazine, carmustin, *cis*platin and tamoxifen. *Melanoma Res* **3**: 127–131

Garbe C, Buttner P, Bertz J, Burg G, D'Hoedt B, Drepper H, Guggenmoos-Holzmann I, Lechner I, Lippold W, Orfanos A, Peters CE, Stadler R and Stroebel W (1995) Primary cutaneous melanoma. I. Identification of prognostic groups and estimation of individual prognosis for 5093 patients. *Cancer* **75**: 2481–2491

Goodall T, Buffey JA, Rennie IG, Benson M, Parsons MA, Faulkner MK and MacNeil S (1994) Effect of melanocyte stimulating hormone on human cultured choroidal melanocytes, uveal melanoma cells and retinal epithelial cells. *Invest Opthal Vis Sci* **35**: 826–837

Gragoudas ES, Egan KM, Seddon JM, Glynn RJ, Walsh SM, Finn SM, Munzennder JE and Spar MD (1991) Survival of patients with metastases from uveal melanoma. *Opthalmology* **98**: 383–390

Gundimeda U, Chen Z-H and Gopalakrishna R (1996) Tamoxifen modulates protein kinase C via oxidative stress in estrogen receptor-negative breast cancer cells. *J Biol Chem* **271**: 13504–13514

Jordan VC, Wolf MF, Mirecki DM, Whitford DA and Welshons WV (1988) Hormone receptor assays: clinical usefulness in the management of carcinoma of the breast. *Crit Rev Clin Lab Sci* **26**: 97–152

Karakousis CP and Driscoll DL (1995) Prognostic parameters in localised melanoma: gender versus anatomical location. *Eur J Cancer* **31**: 320–324

Legha S (1988) Interferon in the treatment of malignant melanoma. *Biother Cancer* **1**: 1–5

Maclay EF, Mastrangelo MJ, Sprandio JD, Bellet RE and Berd D (1989) The importance of tamoxifen to a *cis*platin containing regime in the treatment of metastaticmelanoma. *Cancer* **63**: 1293–1295

Maclay EF, Mastrangelo MJ, Berd D and Bellet RE (1992) Effective combination chemo/hormonal therapy for malignant melanoma: experience with three consecutive trials. *Cancer* **50**: 553–556

MacNeil S, Griffin M, Cooke AM, Pettett NJ, Dawson RA, Owen R and Blackburn GM (1988) Calmodulin antagonists of improved potency and specificity for use in the study of calmodulin biochemistry. *Biochem Pharmacol* **37**: 1717–1723

MacNeil S, Wagner M, Wowk I, Doughty S, Brown J Beaumont J and Blackburn GM (1992) Intracellular regulation of cell adhesion to extracellular matrix components in murine B16 melanoma cells. *Melanoma Res* **2**: 345–354

MacNeil S, Wagner M, Kirkham PR, Blankson EA, Lennard MS, Goodall T and Rennie IG (1993) Inhibition of melanoma cell/matrix interaction by tamoxifen. *Melanoma Res* **3**: 67–74

MacNeil S, Wagner M and Rennie IG (1994) Investigation of the role of signal transduction in attachment to matrix proteins: inhibition of attachment by calmodulin antagonists including tamoxifen. *Clin Exp Metastasis* **12**: 375–384

Markaverich BM, Gregory RR, Alejandro MA, Clark JH and Johnson GA (1988) An endogenous ligand for Type II binding sites. *J Biol Chem* **263**: 7203–7210

Merkel DA and Osborne CK (1988) Use of steroid receptor assays in cancer management. *Rev Endocr Rel Cancer* **30**: 5–12

Mortarini R and Anichini A (1993) From adhesion to signalling: roles of integrins in the biology of human melanoma. *Melanoma Res* **3**: 87–97

Noguchi S, Motowura K, Inaji H, Imaoka S and Koyama H (1993) Down regulation of transforming growth factor-alpha by tamoxifen in human breast cancer. *Cancer* **72**: 131–136

O'Brian CA, Liskamp RM, Solomon DH and Weinstein IB (1985) Inhibition of protein kinase C by tamoxifen. *Cancer Res* **45**: 2462–2465

Paul EV, Parnell BL and Fraker M (1962) Prognosis of malignant melanomas of the choroid and ciliary body. *Int Opthalmol Clin* **2**: 387–402

Piantelli M, Maggiano N, Ricci R, Larocca LM, Capelli A, Scambia G, Isola G, Giorgio Natali P and Ranelletti FO (1995) Tamoxifen and quercetin interact with type II estrogen binding sites and inhibit the growth of human melanoma cells. *J Invest Dermatol* **105**: 248–253

Rajpal S, Moore R and Karakousis CP (1983) Survival in metastatic ocular melanoma. *Cancer* **52**: 334–336

Reintgen D and Saba H (1993) Chemotherapy for stage 4 melanoma: a three year experience with *cis*platin, DTIC, BCNU and tamoxifen. *Semin Surg Oncol* **9**: 251–255

Rayter Z (1991) Steroid receptors in breast cancer. *Br J Surg* **78**: 528–535

Shields JA, Augsberger JJ, Donosco LA, Bernardino VB Jr and Portenar M (1985) Hepatic metastasis and orbital recurrence of uveal melanoma after 42 years. *Am J Opthalmol* **100**: 666–668

Stidham KR, Johnson JL and Seigler HF (1994) Survival superiority of females with melanoma: a multivariate analysis of 6383 patients exploring the significance of gender in prognostic outcome. *Arch Surg* **129**: 316–324

Sutherland RL, Murphy LC, San Foo M, Green MD, Whybourne AM and Krozowski AM (1980) High-affinity anti-oestrogen binding site distinct from the oestrogen receptor. *Nature* **288**: 273–275

Tomlinson S, Macneil S, Walker SW, Ollis CA, Merritt JE and Brown BL (1984) Calmodulin and cell function. *Clin Sci* **66**: 497–508

Van Leeuwen RL, Dekker SK, Arbiser JL, Vermeer BJ, Bruijn JA and Byers HR (1994) Phorbol ester induced rapid attachment and spreading of melanoma cell and the role of extracellular matrix proteins. *Int J Cancer* **57**: 894–900

Vossaert KA, Silverman MK, Kopf AW, Bart RS, Rigal DS, Friedman RJ and Levenstein M (1992) Influence of gender on survival in patients with stage I malignant melanoma. *J Am Acad Dermatol* **26**: 429–440

Walker KJ, Bouzbar N, Robertson J, Ellis IO, Elston CW, Blamey RW, Wilson DW, Griffiths K and Nicholson RI (1988) Immunocytochemical localisation of estrogen receptor in human breast tissue. *Cancer Res* **50**: 3545–3550

 © Cancer Research Campaign 1997